# Clinical characteristics and risk factors for visual prognosis according to the types of infectious endophthalmitis

**DOI:** 10.1371/journal.pone.0278625

**Published:** 2022-12-01

**Authors:** Jae Jung Lee, Yeon Ji Jo, Jong Soo Lee

**Affiliations:** 1 Department of Ophthalmology, Pusan National University School of Medicine, Pusan, Republic of Korea; 2 Biomedical Research Institute, Pusan National University Hospital, Pusan, Republic of Korea; Cairo University Kasr Alainy Faculty of Medicine, EGYPT

## Abstract

**Background:**

Endophthalmitis is a fatal ophthalmological emergency that needs prompt diagnosis and treatment. This study aimed to evaluate the clinical characteristics and investigate risk factors for the visual prognosis of the different types of endophthalmitis.

**Methods:**

This retrospective study included 239 eyes diagnosed with endophthalmitis at the Pusan National University Hospital between January 2006 and December 2020. All patients were classified into six groups based on the etiology of endophthalmitis: post-cataract surgery, post-vitrectomy, post-glaucoma surgery, post-intravitreal injection, endogenous, and post-trauma. Demographics and clinical characteristics such as age, sex, laterality, initial symptoms, the interval between the primary causable event and diagnosis of endophthalmitis, initial and final visual acuity, management, and culture results were reviewed and statistically analyzed. Risk factors for poor visual prognosis were also analyzed according to the type of endophthalmitis.

**Results:**

Of the 239 cases of endophthalmitis, the most common cause was post-cataract surgery, that occurs within two weeks post-surgery. Gram-positive *Staphylococcus* was cultured most frequently. *Fusarium* was characteristically cultured from delayed post-cataract surgery endophthalmitis (14 days–6 weeks post-surgery). Post-vitrectomy endophthalmitis occurred within 3.3 days post-surgery, but post-glaucoma surgery endophthalmitis developed a long period after surgery, averaging 2,742 days. Post-intravitreal injection endophthalmitis occurred most frequently following bevacizumab injection, and *Staphylococcus* was most commonly isolated. For endogenous endophthalmitis, the pyogenic liver abscess was the most common underlying disease, and *Klebsiella* was isolated most frequently. Post-traumatic endophthalmitis mostly occurred in young men. Advanced age and poor initial visual acuity were risk factors for poor visual prognosis (*P* = 0.041, odds ratio = 1.024 and *P* < 0.001, odds ratio = 3.904, respectively, using logistic regression analysis).

**Conclusion:**

Advanced age and initial visual acuity were risk factors for poor visual prognosis in cases of endophthalmitis caused by various etiologies. Early diagnosis and treatment of endophthalmitis are required, especially in older patients.

## Introduction

Endophthalmitis is an inflammatory disease that can induce serious ophthalmological complications. Postoperative endophthalmitis is the most common type (72%), followed by post-traumatic endophthalmitis (20%) and endogenous endophthalmitis (8%) [1, 2].

The visual prognosis of endophthalmitis is related to initial visual acuity (VA), type and pathogenicity of the causative microorganism, time of diagnosis and treatment from the onset of ocular symptoms, treatment method, immune status, and history of the penetrating ocular trauma [3–6]. Endophthalmitis has a poor prognosis, with only 39% of the patients recovering over a VA of 20/400 even with appropriate treatment [4].

Postoperative endophthalmitis is mainly caused by normal bacterial flora in the conjunctiva or external contamination from bacteria invading the eye through a wound or contaminated instrument. The incidence of postoperative endophthalmitis has been reported as 0.05–0.7% [7–9]. The frequency of endophthalmitis and their most common causative microorganisms have been reported to be different among the three types (post-cataract surgery, post-vitrectomy, and post-glaucoma surgery). Endogenous endophthalmitis, which occurs without a history of ocular surgery or trauma, is rare and accounts for 2–15% of all endophthalmitis cases [3, 10]. The incidence of post-traumatic endophthalmitis has been reported as 3.3–17%, and its causative microorganisms are variable [11–13].

Since each type of endophthalmitis has its own clinical characteristics, knowing them can aid in early diagnosis and treatment. Several studies have focused on each type of endophthalmitis, but to the best of our knowledge, no report has classified all the different types according to their etiologies and compared their characteristics. Here, we aimed to compare the clinical characteristics of each type of endophthalmitis, identify the most frequent causative microorganisms of each type, and investigate the risk factors for poor visual prognosis.

## Materials and methods

This retrospective study was approved by the Institutional Review Board of our institute (IRB No. 2205-012-114), and conducted in accordance with the principles outlined in the tenets of the Declaration of Helsinki. Informed consent was waived due to the retrospective nature of this study.

### Study patients

The medical records of 347 patients diagnosed with ‘endophthalmitis’ at Pusan National University Hospital between January 1, 2006, and December 31, 2020, were retrospectively reviewed. Endophthalmitis was diagnosed based on clinical symptoms and signs, such as ocular pain, hyperemia, and hypopyon. Anterior or vitreous samples were collected from all patients suspected of endophthalmitis, and a bacterial or fungal culture test was performed. Treatment was initiated immediately following the diagnosis of endophthalmitis. All patients with endophthalmitis were classified into the following six groups based on etiology: post-cataract surgery, post-vitrectomy, post-glaucoma surgery, post-intravitreal injection, endogenous, and post-trauma.

Demographic and clinical characteristics such as age, sex, laterality, initial symptoms, interval between the primary causable event and diagnosis of endophthalmitis, initial VA, final VA, treatment method, and culture results were investigated, and the differences between each group were compared. VA was converted to the logarithm of the minimal angle of resolution (LogMAR) for statistical analysis. Finger count was defined as 2.301, hand movement as 2.602, light perception as 2.903, and no light perception as 3.204 according to the study by Kaneko et al. [14].

Further investigation was performed for each group. Post-cataract surgery endophthalmitis was classified into the following three groups according to symptom onset: acute (< 14 days), delayed (14 days–6 weeks), and chronic (> 6 weeks). The type of vitreoretinal disease was investigated in post-vitrectomy endophthalmitis, and the type of glaucoma surgery was investigated in post-glaucoma surgery endophthalmitis. The type of intravitreal drug was investigated in post-intravitreal injection endophthalmitis, and underlying disease was investigated in endogenous endophthalmitis. In post-traumatic endophthalmitis, the form of trauma was classified into ocular perforation or intraocular foreign body.

We defined poor visual prognosis as the final VA with LogMAR 1.0, and the factors related to poor visual prognosis were analyzed.

### Statistical analysis

All statistical analyses were performed using IBM SPSS Statistics for Windows, Version 21.0 (IBM Corp., Armonk, NY, USA). The Kruskal–Wallis test with post-hoc Mann–Whitney U test was used to compare continuous variables and the one-way analysis of variance (ANOVA) with Tukey post-hoc test was used to compare categorical variables among the six groups. Multiple comparisons were made using the Mann–Whitney U test with Bonferroni correction; *P*-value < 0.0033 was considered statistically significant according to an alpha value of 15. For categorical variables, Tukey’s multiple comparison test was used; *P*-value < 0.05 was considered statistically significant. For comparison between initial and final VAs, paired t-test or Wilcoxon signed-rank test was used depending on the distribution of the data. Univariate and multivariate logistic regression analyses were used to analyze risk factors associated with poor visual prognosis. A *P*-value < 0.05 was considered statistically significant.

## Results

**Fig 1:**
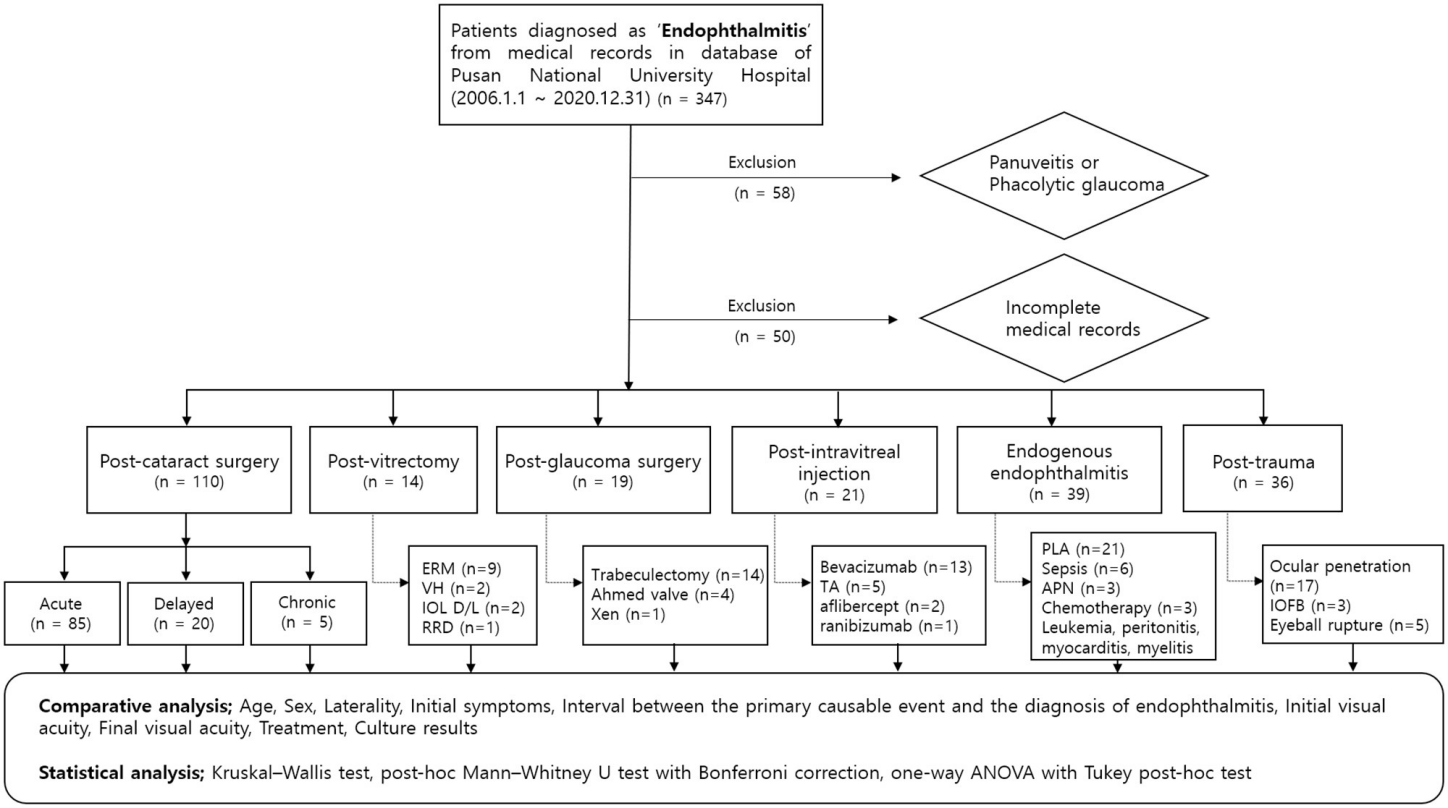
Flow chart of endophthalmitis classification according to etiology. (APN: acute pyelonephritis, ERM: epiretinal membrane, IOFB: intraocular foreign body, IOL D/L: intraocular lens dislocation. PLA: pyogenic liver abscess, RRD: rhegmatogenous retinal detachment, TA: triamcinolone acetonide, and VH: vitreous hemorrhage).

A total of 347 patients who attended Pusan National University Hospital between January 1, 2006, and December 31, 2020, were diagnosed with endophthalmitis. Among them, 58 patients were excluded because they were misdiagnosed with endophthalmitis, including uveitis or phacolytic glaucoma. Fifty patients were excluded because of incomplete medical records. Finally, 239 patients (239 eyes with endophthalmitis) were enrolled in this study (Fig 1).

A total of 134 (56.1%) patients were men and 105 (43.9%) were women. The mean age was 69.0 ± 12.5 years. Right, and left eye involvement was observed in 115 (48.1%) and 124 patients (51.9%), respectively. Demographic data are summarized in Table 1.

**Table 1:** Demographics of patients with infectious endophthalmitis.

Parameters		Results
Age (years)		69.0 ± 12.5
Gender (Men: Women)		134: 105
Initial BCVA (logMAR)		2.16 ± 0.87
Initial symptoms	Decreased vision	117 (49.0%)
	Ocular pain	79 (33.1%)
	Hypopyon	20 (8.4%)
	Hyperemia	20 (8.4%)
	Floaters	3 (1.3%)
Type of endophthalmitis	Post-cataract surgery	110 (46.0%)
	Post-vitrectomy	14 (5.9%)
	Post-glaucoma surgery	19 (7.9%)
	Post-intravitreal injection	21 (8.8%)
	Endogenous	39 (16.3%)
	Post-trauma	36 (15.1%)

BCVA = best-corrected visual acuity; logMAR = logarithm of the minimal angle of resolution

Decreased vision (117 eyes, 49.0%) was the most common initial symptom, followed by ocular pain (79 eyes, 33.1%). Hyperemia (20 eyes, 8.4%), hypopyon (20 eyes, 8.4%), and floaters (3 eyes, 1.3%) were also noted (Table 1).

The positive bacterial culture rate was 41.8%. Gram-positive *Staphylococcus*, which was the most common, was detected in 29 eyes (12.1%), followed by gram-positive *Streptococcus* (23 eyes, 9.6%). Gram-negative *Klebsiella* was detected in 17 eyes (7.1%), and all the cases were cultured from endogenous endophthalmitis (Table 2).

**Table 2:** Microorganisms of patients with infectious endophthalmitis.

Microorganism		number
No growth		139 (58.2%)
Gram positive	*Staphylococcus*	29 (12.1%)
	*Streptococcus*	23 (9.6%)
	*Enterococcus*	12 (5.0%)
	*Actinomyces*	1 (0.4%)
Gram negative	*Pseudomonas*	4 (1.7%)
	*Klebsiella*	17 (7.1%)
	*Achromobacter*	2 (0.8%)
	*Enterobacter*	3 (1.2%)
	*Haemophilus*	2 (0.8%)
Fungus	*Fusarium*	4 (1.6%)
	*Candida*	2 (0.8%)
	*Aspergillus*	1 (0.4%)

Vitrectomy (175 eyes, 73.2%) was the most common treatment method, followed by intravitreal antibiotic injection (57 eyes, 23.8%). Evisceration or enucleation was performed in seven eyes (2.9%). There was no significant difference between the final VA of the vitrectomy and intravitreal antibiotics injection groups (*P* = 0.753, paired t-test).

### Post-cataract surgery endophthalmitis

The mean age of patients in this group was 73.4 ± 9.5 years, 50 patients (45.5%) were men and 60 (54.5%) were women. A total of 54 (49.1%) and 56 patients (50.9%) had right and left eye involvement, respectively. More than half of the patients (59 eyes, 53.6%) visited the ophthalmology department because of decreased VA. Ocular pain (30 eyes, 27.2%) and hypopyon (14 eyes, 12.7%) were the most common symptoms. A total of 110 patients were classified into the following three groups according to the onset time of symptoms: acute (< 14 days), delayed (14 days–6 weeks), and chronic (> 6 weeks). Acute endophthalmitis (85 eyes, 77.3%) was the most common group, and patients were diagnosed with endophthalmitis 4.0 ± 2.9 days after cataract surgery. Delayed endophthalmitis occurred in 20 eyes (18.2%), and the initial symptoms appeared 25.4 ± 10.9 days after surgery. Chronic endophthalmitis (5 eyes, 4.5%) was diagnosed 245.0 ± 282.2 days after surgery. The positive bacterial culture rate was 38.8%. Gram-positive *Staphylococcus* was detected in 10 eyes, *Enterococcus* in nine, and *Streptococcus* in eight. However, the fungus *Fusarium* was found in all culture-positive cases of delayed endophthalmitis. In chronic endophthalmitis, gram-positive *Actinomyces* was cultured in one case. There was significant VA improvement from 2.18 ± 0.76 LogMAR to 1.18 ± 1.11 LogMAR following the treatment of acute endophthalmitis (*P* < 0.001, paired t-test). However, there was no significant change after the treatment of delayed and chronic endophthalmitis (*P* = 0.285 and *P* = 0.168, respectively; Wilcoxon signed-rank test). Vitrectomy was performed in 81.8% of the cases, and there were no eyeball losses. There were no statistical differences in age, sex, laterality, initial symptoms, final VA, and positive culture rate among the three groups. Delayed endophthalmitis showed better initial VA than that shown by acute endophthalmitis (*P* < 0.001, post-hoc Mann–Whitney U test). Intravitreal antibiotic injection was more frequent in patients with chronic endophthalmitis than those with acute and delayed endophthalmitis, and vitrectomy was performed in 95% of delayed endophthalmitis cases. The difference between treatment methods was statistically significant (*P* = 0.012, Tukey post-hoc test; Table 3).

**Table 3:** Comparative analysis of endophthalmitis after cataract surgery according to onset time.

	A. acute (n = 85)	B. delayed (n = 20)	C. chronic (n = 5)	A-C *P*-value	A vs B *P*-value	A vs C *P*-value	B vs C *P*-value
Age (years)	73.9 ± 9.3	72.9 ± 10.0	67.6 ± 9.7	0.406^a^	0.180^b^	0.893^b^	0.272^b^
Gender (Men: Women)	37:48	9:11	4:1	0.287^c^	0.992^d^	0.255^d^	0.343^d^
Laterality (Right: Left)	42:43	10:10	2:3	0.918^c^	0.999^d^	0.914^d^	0.918^d^
Initial symptoms (1:2:3:4:5)	3:23:12:45:2	1:7:1:11:0	1:0:1:3:0	0.868^c^	0.856^d^	0.997^d^	0.979^d^
Initial visual acuity (logMAR)	2.18 ± 0.76	1.20 ± 1.11	1.18 ± 1.07	<0.001^a^*	0.053^b^	<0.001 ^b^**	0.767^b^
Final visual acuity (logMAR)	1.18 ± 1.11	0.91 ± 1.22	1.78 ± 1.55	0.332^a^	0.530^b^	0.169^b^	0.488^b^
Treatment (injection: vitrectomy)	16:69	1:19	3:2	0.016^c^*	0.305^d^	0.050^d^	0.012^d^*
Culture (x:o)	52:33	17:3	4:1	0.104^c^	0.107^d^	0.659^d^	0.975^d^

logMAR = logarithm of the minimal angle of resolution, acute: < 14 days, delayed: 14 days– 6 weeks, chronic: > 6 weeks

1: hyperemia, 2: ocular pain, 3: hypopyon, 4: decreased vision, 5: floaters

^a^The *P*-value was obtained from the Kruskal–Wallis test.

^b^The *P*-value was obtained from the post-hoc test (Bonferroni).

^c^The *P*-value was obtained from the one-way ANOVA test.

^d^The *P*-value was obtained from the Tukey post-hoc test.

*P-value < 0.05 was considered statistically significant.

**P-value < 0.0167 was considered statistically significant (alpha value of 3).

### Post-vitrectomy endophthalmitis

**Fig 2:**
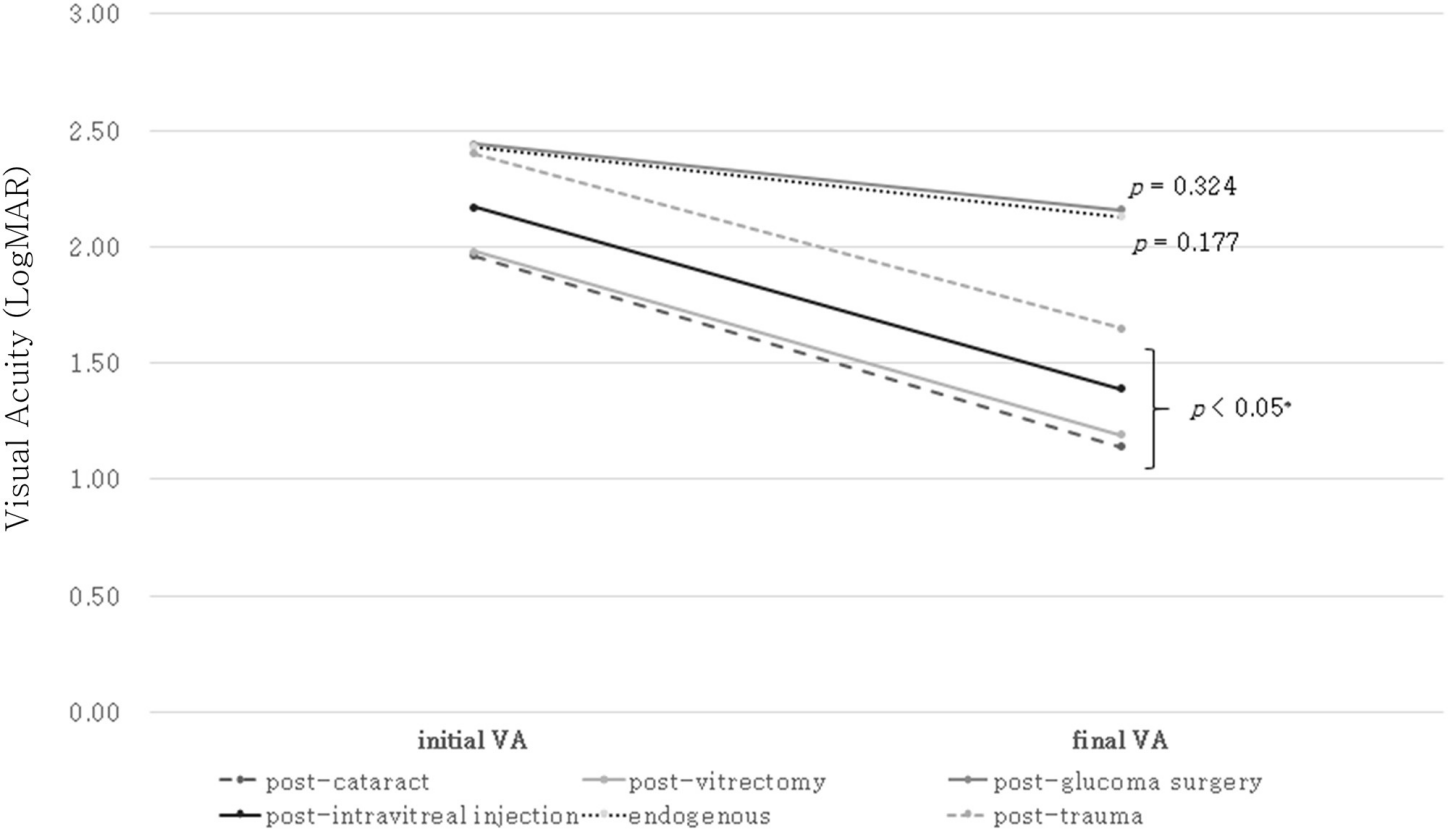
Comparison between initial visual acuity and final visual acuity. Final visual acuity improved after treatment in the post-cataract surgery, post-vitrectomy, post-intravitreal injection, and post-traumatic endophthalmitis groups. However, there was no significant change between initial and final visual acuity in the post-glaucoma surgery and endogenous endophthalmitis groups.

The presence of an epiretinal membrane (nine eyes, 64.3%) was the most common cause of vitrectomy. Vitreous hemorrhage (two eyes, 14.3%), intraocular lens dislocation (two eyes, 14.3%), and retinal detachment (one eye, 7.1%) were also observed. Combined surgery (vitrectomy with cataract surgery) was performed in eight eyes (57.1%), and vitrectomy alone was performed in six eyes (42.9%). A 23G pars plana vitrectomy was performed in six eyes (42.9%), and a 25G was used in eight eyes (57.1%). Sutureless vitrectomy was performed in eight eyes (57.1%). Ocular pain (seven eyes, 50.0%) was the most common initial symptom, followed by decreased VA (five eyes, 35.7%). Symptoms of endophthalmitis appeared on an average of 3.3 ± 2.9 days after surgery. The positive bacterial culture rate was 35.7%, and gram-positive *Staphylococcus* was the most common bacteria (four eyes, 28.6%). VA significantly improved after treatment, from 1.97 ± 0.84 LogMAR to 1.19 ± 1.27 LogMAR (*P* = 0.017, Wilcoxon signed-rank test, Fig 2). Vitrectomy was performed in 85.7% (12 eyes) cases, and two patients were treated with an intravitreal antibiotic injection. The other demographic data are shown in Table 4.

**Table 4:** Comparative analysis of endophthalmitis according to its causes or types.

	A. post-cataract (n = 110)	B. post-vitrectomy (n = 14)	C. post-glaucoma (n = 19)	D. post-injection (n = 21)	E. endogenous (n = 39)	F. post-trauma (n = 36)	A-F *P*-value
Age (years)	73.4 ± 9.5	65.1 ± 10.8	68.6 ± 11.6	68.9 ± 11.5	70.1 ± 12.7	55.9 ± 13.0	<0.001^a^*
Gender (Men: Women)	50:60	4:10	12:7	8:13	27:12	33:3	<0.001^b^*
Laterality (Right: Left)	54:56	6:8	7:12	11:10	20:19	17:19	0.917^b^
Initial symptoms (1:2:3:4:5)	3:30:14:59:2	1:7:0:5:1	4:12:3:0:0	2:7:2:10:0	6:8:1:24:0	2:15:0:19:0	<0.001^b^*
Interval between the primary causable event and diagnosis of endophthalmitis	18.8 ± 74.0	3.3 ± 2.9	2741.8 ± 4009.8	5.6 ± 5.5	15.9 ± 29.2	6.8 ± 21.7	<0.001^a^*
Initial visual acuity (logMAR)	1.96 ± 0.94	1.97 ± 0.84	2.44 ± 0.85	2.17 ± 0.78	2.43 ± 0.68	2.40 ± 0.82	0.005^a^*
Final visual acuity (logMAR)	1.16 ± 1.15	1.19 ± 1.27	2.16 ± 1.14	1.34 ± 1.01	2.12 ± 1.27	1.62 ± 1.39	0.001^a^*
Treatment (injection: vitrectomy:evisceration)	20:90:0	2:12:0	6:12:1	8:13:0	16:21:2	5:27:4	0.024^b^*
Culture (x:o)	73:37	9:5	8:11	13:8	13:26	23:13	0.007^b^*

logMAR = logarithm of the minimal angle of resolution

1: hyperemia, 2: ocular pain, 3: hypopyon, 4: decreased vision, 5: floaters

^a^The *P*-value was obtained from the Kruskal–Wallis test.

^b^The *P*-value was obtained from the one-way ANOVA test.

*P-value < 0.05 was considered statistically significant.

### Post-glaucoma surgery endophthalmitis

Trabeculectomy (14 eyes, 73.7%) was the most common surgery, followed by Ahmed valve implantation (4 eyes, 21.1%) and one case of Xen stent insertion. Mitomycin C (MMC) was used in all cases of trabeculectomy and Xen stent insertion. Regarding endophthalmitis after trabeculectomy, two eyes developed endophthalmitis immediately after surgery, and 12 eyes developed endophthalmitis owing to bleb leakage. Endophthalmitis secondary to Ahmed valve exposure occurred in three eyes, and one eye developed endophthalmitis immediately after Ahmed valve implantation. Xen-related endophthalmitis occurred from Xen stent exposure. Ocular pain (12 eyes, 63.2%) was the most common initial symptom, followed by hyperemia (four eyes, 21.1%) and hypopyon (three eyes, 15.8%). Endophthalmitis symptoms appeared 2741.8 ± 4009.8 days after surgery, which was a wide range. The positive bacterial culture rate was 57.9%, and gram-positive *Streptococcus* was the most common (eight eyes). There was no significant change in final VA from 2.43 ± 0.85 LogMAR to 2.16 ± 1.14 LogMAR (*P* = 0.324, Fig 2). Vitrectomy was performed in 63.2% (12 eyes) of cases, six patients were treated with intravitreal antibiotics injection, and one patient lost an eye. The other demographic data are shown in Table 4.

### Post-intravitreal injection endophthalmitis

Bevacizumab (Avastin; Genentech, South San Francisco, CA, USA) was the most common causative agent of post-intravitreal injection endophthalmitis (13 eyes, 61.9%). The incidence of endophthalmitis after bevacizumab injection was 0.070% (13/18,622) in our cohort. Triamcinolone acetonide (TA, Tamceton, Hanall, BioPharma) was used in 5 eyes (23.8%), and the incidence of endophthalmitis after TA was 0.065% (5/7,637). Aflibercept (Eylea; Regeneron Pharmaceuticals, Inc, Tarrytown, NY, USA) was used in 2 eyes (9.5%), and the incidence of endophthalmitis after aflibercept injection was 0.022% (2/9,126). Ranibizumab (Lucentis; Genentech) was used in 1 eye (4.8%), and the incidence of endophthalmitis after ranibizumab injection was 0.010% (1/10,426). Decreased VA (10 eyes, 47.6%) was the most common initial symptom. Ocular pain (seven eyes, 33.3%), hypopyon (two eyes, 9.5%), and hyperemia (two eyes, 9.5%) were also noted. Endophthalmitis appeared 5.6 ± 5.5 days after intravitreal injection. The bacterial culture-positive rate was 38.1%. Gram-positive *Staphylococcus* was cultured in six eyes (28.6%), and *Streptococcus* was cultured in two eyes (9.5%). VA significantly improved after treatment, from 2.17 ± 0.78 LogMAR to 1.34 ± 1.01 LogMAR (*P* = 0.002, Fig 2). Vitrectomy was performed in 61.9% (13 eyes) of the cases, and eight patients (38.1%) were treated with intravitreal antibiotics injection. The other demographic data are shown in Table 4.

### Endogenous endophthalmitis

Pyogenic liver abscess (21 patients, 53.8%) was the most common underlying disease. Sepsis (6 patients, 15.4%), acute pyelonephritis (3 patients, 7.7%), chemotherapy (3 patients, 7.7%), acute leukemia (1 patient, 2.6%), peritonitis (1 patient, 2.6%), myocarditis (1 patient, 2.6%), and pyogenic myelitis (1 patient, 2.6%) were also present. Two patients did not have an infective focus other than the eye, one had diabetes mellitus, and the other was undergoing hemodialysis. Diabetes mellitus was found in 56.4% of the cases. The most common initial symptom was decreased VA (24 eyes, 61.5%), followed by ocular pain (eight eyes, 20.5%), hyperemia (six eyes, 15.4%), and hypopyon (one eye, 2.6%). Endophthalmitis symptoms appeared on an average of 15.9 ± 29.2 days after the onset of other infections. The positive bacterial culture rate was 66.7%, and gram-negative *Klebsiella* was cultured in 17 eyes (43.6%). *Streptococcus* (4 eyes, 10.3%), *Staphylococcus* (1 eye, 2.6%), *Enterococcus* (1 eye, 2.6%), and *Pseudomonas* (1 eye, 2.6%) were also detected. The fungus *Candida* was cultured in two eyes (5.1%). Final VA did not change significantly from 2.43 ± 0.68 LogMAR to 2.12 ± 1.27 LogMAR (*P* = 0.177, Fig 2). Vitrectomy was performed in 53.8% (21 eyes) of the cases, 16 patients were treated with intravitreal antibiotics injection, and evisceration was performed in two patients. The other demographic data are shown in Table 4.

### Post-traumatic endophthalmitis

Ocular perforation (17 eyes, 47.2%) was the most common type of ocular trauma. The eyeball of seven patients was perforated by a tree branch, five were perforated by an iron wire, and five were perforated by an iron nail. Intraocular foreign body (14 eyes, 38.9%) was the next common type of ocular trauma. An iron piece was the most common foreign body (12 eyes). One patient presented with a stone in the eye, and another with a plastic object in the eye. Eyeball rupture occurred in five cases (13.9%). The mean age was 55.9 ± 13.0 years, which was lower than that of other groups. Most of the cases were men (33 patients, 91.6%), and in 17 patients, the right eye was involved (47.2%). Decreased VA (19 eyes, 52.8%) was the most common symptom, followed by ocular pain (15 eyes, 41.7%) and hyperemia (2 eyes, 5.6%). Endophthalmitis symptoms appeared 6.8 ± 21.7 days after ocular trauma, but one case was detected 120 days after trauma following the removal of an intraocular foreign body. The positive bacterial culture rate was 36.1%, and gram-positive *Staphylococcus* was cultured in eight eyes (22.2%). *Streptococcus*, *Enterobacter*, and *Aspergillus* were also cultured. VA significantly improved after treatment, from 2.63 ± 0.82 LogMAR to 1.54 ± 1.39 LogMAR (*P* = 0.002, Fig 2). Vitrectomy was performed in 75.0% (27 eyes) of the cases, five patients were treated with intravitreal antibiotics injection, and four patients (11.1%) suffered eye loss.

To summarize the comparison results in this study, sex, age, laterality, initial symptoms, the interval between the primary causable event and diagnosis of endophthalmitis, initial VA, final VA, management, and culture results were analyzed among six groups. There were statistically significant differences in all parameters except for laterality among the six groups (Kruskal-Wallis test and one-way ANOVA test, Table 4).

A post-hoc Mann–Whitney U test with Bonferroni correction (*P*-value < 0.0033) was used for continuous variables, such as age, the interval between the primary causable event and diagnosis of endophthalmitis, initial VA, and final VA. The mean age of post-traumatic endophthalmitis was significantly lower than that in post-cataract surgery, post-glaucoma, post-intravitreal injection, and endogenous endophthalmitis (*P* < 0.001, for all). For post-glaucoma surgery endophthalmitis, the interval between the primary causable event and diagnosis of endophthalmitis was significantly longer than that in other types of endophthalmitis (*P* < 0.001). For post-traumatic endophthalmitis, the interval between the primary causable event and diagnosis of endophthalmitis was significantly shorter than that in post-cataract surgery and endogenous endophthalmitis, and longer than that in post-injection endophthalmitis (*P* < 0.001, for all). The final VA in post-cataract surgery endophthalmitis was significantly better than that in post-glaucoma surgery and endogenous endophthalmitis (*P* < 0.001). These statistical results are shown in Table 5.

**Table 5:** Post-hoc analysis of results in Table 4.

	*P*-value														
	A-B	A-C	A-D	A-E	A-F	B-C	B-D	B-E	B-F	C-D	C-E	C-F	D-E	D-F	E-F
Age (years)^a^	0.006	0.049	0.044	0.163	<0.001*	0.412	0.495	0.238	0.008	0.957	0.579	0.001*	0.625	<0.001*	<0.001*
Gender (Male: Female) ^b^	0.797	0.645	0.986	0.086	<0.001**	0.287	0.991	0.061	<0.001**	0.534	0.997	0.262	0.138	0.001**	0.299
Laterality (Right: Left) ^b^	0.998	0.925	0.784	0.816	0.847	0.999	0.994	0.995	0.784	0.926	0.910	0.979	0.936	0.999	0.999
Initial symptoms (1:2:3:4:5)^b^	0.741	<0.001**	0.809	0.962	0.774	0.134	0.791	0.974	0.998	0.030**	0.001**	0.001**	0.995	0.868	0.998
Interval between the primary causable event and diagnosis of endophthalmitis^a^	0.019	<0.001**	0.566	0.659	<0.001**	<0.001**	0.061	0.078	0.485	<0.001**	<0.001**	<0.001**	0.975	<0.001**	0.001**
Initial visual acuity (logMAR)^a^	0.894	0.011	0.615	0.006	0.006	0.098	0.778	0.095	0.117	0.069	0.585	0.706	0.109	0.081	0.822
Final visual acuity (logMAR)^a^	0.852	<0.001**	0.176	<0.001**	0.190	0.032	0.454	0.064	0.622	0.012	0.884	0.102	0.023	0.634	0.110
Treatment (injection: vitrectomy:evisceration) ^b^	0.769	0.982	0.472	0.324	0.520	0.978	0.677	0.673	0.970	0.968	0.977	0.481	0.862	0.068	0.028**
Culture (x:o) ^b^	0.880	0.333	0.999	0.004**	0.790	0.783	0.886	0.041**	0.979	0.788	0.987	0.605	0.030**	0.881	0.072

A: post-cataract, B: post-vitrectomy, C: post-glaucoma, D: post-injection, E: endogenous, F: post-trauma

logMAR = logarithm of the minimal angle of resolution

1: hyperemia, 2: ocular pain, 3: hypopyon, 4: decreased vision, 5: floaters

^a^The *P*-value was obtained from the post-hoc Mann–Whitney U test with Bonferroni correction (*P*-value < 0.0033)

^b^The *P*-value was obtained from the Tukey post-hoc test (*P*-value < 0.05).

*P-value < 0.0033 was considered statistically significant.

**P-value < 0.05 was considered statistically significant.

Tukey’s post-hoc test was used for categorical variables such as sex, initial symptoms, management, and culture results. Men were predominant in the post-traumatic endophthalmitis group compared with the post-cataract surgery, post-vitrectomy, and post-injection endophthalmitis groups (*P* < 0.05, for all). Initial symptoms were statistically significantly different between the post-glaucoma surgery endophthalmitis group and the other four endophthalmitis groups (post-cataract, post-injection, endogenous, and post-traumatic endophthalmitis; *P* < 0.05, for all). The treatment method for endogenous endophthalmitis and post-traumatic endophthalmitis was statistically significantly different (*P* = 0.024). The positive bacterial culture rate was significantly higher in endogenous endophthalmitis than that in post-cataract surgery, post-vitrectomy, and post-injection endophthalmitis (*P* < 0.05, for all). These statistical results are shown in Table 5.

We defined poor visual prognosis as a final VA of 1.0 LogMAR and analyzed the risk factors for poor visual prognosis. In the univariate logistic regression analysis, age (*P* = 0.027, odds ratio [OR] = 1.024) and initial VA (*P* < 0.001, OR = 3.883) were identified as risk factors for poor visual prognosis. In the multivariate logistic regression analysis, age (*P* = 0.041, OR = 1.024) and initial VA (*P* < 0.001, OR = 3.904) were identified as risk factors. Older patients developed poorer visual prognoses, and a lower initial VA led to poorer visual prognoses (Table 6).

**Table 6:** Univariate and multivariate logistic regression analyses of risk factors associated with poor final visual acuity.

Variables	Odds ratio	95.0% Confidence interval	*P*-value
Univariate analysis			
Age	1.024	1.003–1.046	0.027
Initial visual acuity	3.883	2.520–5.984	<0.001
Multivariate analysis			
Age	1.024	1.001–1.048	0.041
Initial visual acuity	3.904	2.520–6.047	<0.001

‘Poor final visual acuity’ defined as 1.0 logarithm of the minimum angle of resolution.

*P-value < 0.05 was considered statistically significant.

## Discussion

Endophthalmitis can be diagnosed based on symptoms such as ocular pain, decreased VA, hyperemia, and signs such as anterior chamber inflammation, hypopyon, and vitreous haziness. These are similar to uveitis, and some uveitis cases can be misdiagnosed as endophthalmitis. In our study, 58 patients with uveitis were misdiagnosed with endophthalmitis; therefore, differential diagnosis is very important. Symptoms of endophthalmitis are easily diagnosed in postoperative endophthalmitis, but early diagnosis is difficult in endogenous cases, implying that suspecting endophthalmitis is crucial in such cases.

The frequency of endophthalmitis after cataract surgery has been reported as 0.04–0.2% but has been gradually decreasing because of improved surgical instruments and prophylactic antibiotic use [9, 15, 16]. However, the incidence of post-cataract surgery endophthalmitis was high in this study because of the high frequency of cataract surgery. Most endophthalmitis cases occurred 3–5 days after surgery, but chronic endophthalmitis occurred 6 weeks after surgery [16, 17]. Therefore, we classified patients with post-cataract surgery endophthalmitis into three groups based on the onset of symptoms. *Staphylococcus* was the most common microorganism, a finding similar to that in other studies [18–20].

*Fusarium*, although uncommon, appeared abnormally in 2020, and accounts for 80% of post-cataract surgery endophthalmitis cases in 2020, in this study. Additionally, *Fusarium* was mostly cultured in delayed endophthalmitis, owing to the endophthalmitis outbreak from contaminated viscoelastic materials in South Korea in 2020. The final VA of endophthalmitis after cataract surgery was significantly better than that of endogenous endophthalmitis and post-glaucoma surgery endophthalmitis, which is consistent with the findings of a study by Kim et al. [21] and Chung and Ham [22]. However, their studies included only a small number of patients than did our study, which analyzed 239 eyes. Lee and Park [23] showed results different from that of ours, reporting that the visual prognosis of post-glaucoma surgery endophthalmitis was the best in 59 eyes. The statistical reliability of their study was low as they replaced no light perception, light perception, and hand movement with 0, and they did not convert VA to LogMAR.

There was a high incidence rate of endophthalmitis with the early use of 20G vitrectomy [24–26]. The incidence of endophthalmitis decreased to 0.01–0.051% since the introduction of 23G or 25G microincision vitrectomy [7, 9]. Recently, 23G or 25G sutureless microincision vitrectomy has been widely performed. Regarding sutureless vitrectomy, the risk of endophthalmitis may increase because of leakage or vitreous incarceration through the sclerotomy site [27]. In this study, the incidence of endophthalmitis after vitrectomy was 5.9%, which was the lowest among all the endophthalmitis types. The proportion of sutureless vitrectomy was not particularly high in this study. Similar to findings from other studies, gram-positive *Staphylococcus* was the most common causative bacteria [28]. There have been several reports of poor visual prognosis of post-vitrectomy endophthalmitis after treatment [25, 29]. However, VA significantly improved after treatment and, compared to other endophthalmitis types, the poor visual prognosis was not observed in our study.

The incidence of post-glaucoma surgery endophthalmitis has been reported as 0.061–9.6% [7, 30–32]. Trabeculectomy without the use of anti-metabolites such as 5-fluorouracil (5-FU) or MMC demonstrated a lower incidence of endophthalmitis [31, 33–35]. This is because anti-metabolite use generated cystic avascular blebs susceptible to infection [20, 36]. The direct infiltration of bacteria through the thin conjunctiva of the bleb is the main route of infection, which is different from that of other postoperative endophthalmitis. Because of this unique route of infection, the onset period of endophthalmitis varies from months to years [30, 31, 37]. In this study, endophthalmitis symptoms occurred 2741.8 ± 4009.8 days after surgery. *Streptococcus* and *Hemophilus influenza* are known to be the main causative bacteria for bleb-related endophthalmitis [18, 20, 38]. Gram-positive *Streptococcus* was the most common bacterium in this study, a finding similar to that of other studies. VA did not significantly improve after treatment because the initial VA was low owing to advanced glaucoma. The risk factors for post-glaucoma surgery endophthalmitis included thin bleb, contact lens use, 5-FU eye drops, MMC application during surgery, conjunctivitis, and ocular trauma. Therefore, patients with risk factors for endophthalmitis should be carefully examined to prevent its occurrence [31, 35, 39].

Post-intravitreal injection endophthalmitis can occur with the use of various drugs such as bevacizumab, ranibizumab, aflibercept, and triamcinolone acetonide (TA). The incidence of endophthalmitis following intravitreal anti-vascular endothelial growth factor (anti-VEGF) injection was reported as 0.029–0.15% [40, 41] and following intravitreal TA injection was reported as 0.030–0.87% [42, 43]. Bevacizumab, a US FDA-approved treatment for colorectal cancer, is used as an off-label drug, and there is a possibility of contamination in the process of dispensing the drug for intravitreal use [44]. In this study, the highest incidence of endophthalmitis after bevacizumab injection was 0.070%, followed by TA (0.065%). An animal experiment demonstrated that TA increases susceptibility to infection by lowering the threshold of bacteria through the induction of localized intraocular immune suppression [45]. Additionally, inflammatory reactions to chemicals such as benzyl alcohol included in TA can occur [44]. Non-infectious endophthalmitis after the injection has been frequently reported after aflibercept use [46, 47]. It is characterized by slight ocular pain, no hypopyon, and a good response to steroid treatment [47]. We excluded all cases of non-infectious endophthalmitis in this study, and infectious endophthalmitis after intravitreal injection occurred in 9.5% of the cases.

Endogenous endophthalmitis is caused by the hematogenous spread of pathogens from distant infectious loci in the body [48]. Immunocompromised patients or those with diabetes mellitus, liver abscess, and drug abuse are susceptible to endogenous endophthalmitis [49]. The incidence was reported as 0.005–1.92% [50–52]. Endogenous endophthalmitis is rare, accounting for 2–15% of all endophthalmitis types. However, endogenous endophthalmitis accounted for 16.3% of this study, which was relatively high as it was performed in a tertiary referral hospital. Gram-negative bacteria are common in endogenous endophthalmitis. *Klebsiella pneumonia* is the most common species associated with pyogenic liver abscess [53, 54]. Endogenous endophthalmitis caused by *Klebsiella pneumonia* has been reported in poor visual prognosis [55, 56]. In this study, endophthalmitis caused by *Klebsiella* accounted for 43.6% of the total endogenous endophthalmitis cases, and the visual prognosis was poor. The positive bacterial culture rate was higher than that in other types of endophthalmitis, which is considered as the highest virulence of the bacteria, and is related to poor visual prognosis.

The incidence of post-traumatic endophthalmitis has been reported as 3.3–17% [11–13], and it accounts for 25–30% of total endophthalmitis cases [57]. Post-traumatic endophthalmitis occurred mainly in young men owing to the high incidence of trauma. In post-traumatic endophthalmitis, the causative microorganisms, cause of trauma, and regional characteristics vary such that there is no established treatment protocol. Additionally, post-traumatic endophthalmitis was excluded from the Endophthalmitis Vitrectomy Study (EVS) [5]. Early diagnosis of post-traumatic endophthalmitis can be difficult because infective signs may be obscured by hemorrhage and structural damage. Known risk factors for post-traumatic endophthalmitis in previous studies included intraocular foreign bodies, delayed primary closure and systemic antibiotic administration, injury in a rural area, and lens capsule damage [11, 12, 58]. Therefore, patients with known risk factors should be carefully examined. *Staphylococcus epidermidis* and *Bacillus cereus* are common species [38], and *Staphylococcus* was the most common microorganism in our study.

There have been several comparative analyses of endophthalmitis according to etiology. Kim et al. [21] analyzed endophthalmitis as either exogenous or endogenous. Exogenous endophthalmitis comprised post-cataract, post-traumatic, and bleb-related endophthalmitis. Post-cataract surgery endophthalmitis was the most common type and the visual prognosis was reported to be the best in their study. VA did not improve in bleb-related endophthalmitis, and in cases of endogenous endophthalmitis, VA significantly improved after treatment, but the final visual prognosis was poor. In our study, we performed additional analysis of post-vitrectomy and post-intravitreal injection endophthalmitis. Contrary to Kim et al.’s study findings, our study showed no significant VA improvement in both post-glaucoma surgery and endogenous endophthalmitis. This is because the initial VA of the post-glaucoma surgery endophthalmitis was low due to advanced glaucoma, and the diagnosis of endogenous endophthalmitis was delayed because of other systemic problems. In this study, low initial VA is reported as a risk factor for poor visual prognosis, which is consistent with the findings of EVS and Lee and Park’s study [5, 23].

According to the EVS, vitrectomy is as an effective treatment for an initial VA lower than light perception [5]. However, there were several reports that early vitrectomy is better for visual prognosis through improved vitrectomy instruments and surgeon skill [21, 59]. Contrastingly, some reports have shown no significant difference in final VA between vitrectomy and the use of intravitreal antibiotics injection [60, 61]. Kim et al. reported that there was no significant difference between vitrectomy and use of intravitreal antibiotics injection when the initial VA was better than hand movement; thus, intravitreal antibiotics injection can be a primary treatment for endophthalmitis [62]. Furthermore, repeated intravitreal antibiotics injection can be a useful treatment for resistant types of endophthalmitis [63]. In this study, we observed that intravitreal antibiotics injection could be an effective treatment for endophthalmitis, as reported by Kim et al. [62].

The positive bacterial culture rate was 41.8% in this study, which was lower than that observed in the EVS (69%) [5] and the studies by Kim et al. (68.3%) [21] and Lee and Park (63%) [23]. This was owing to the change in the bacterial culture method in our hospital, which influenced the results. In August 2011, the culture method was changed from the conventional culture method that uses blood and chocolate agar plates to BacT/Alert PF (bioMérieux, Marcy l’Etoile, France) pediatric culture bottles. The positive bacterial culture rate of the former method was 33.3% and that in the latter method using pediatric culture bottles was 60.7% [64]. Previous studies have reported *Staphylococcus epidermidis* as the most common causative microorganism for endophthalmitis and *Klebsiella pneumoni*a as the most common bacteria for endogenous endophthalmitis [21, 54, 65, 66]. Our results were consistent with those in previous reports.

This study has several limitations. It used a retrospective design; therefore, there is a possibility of selection bias. However, the data for this study were collected consecutively over a long period, of approximately 15 years. In addition, there was no specific protocol or methodology for choosing the treatment method owing to the retrospective nature of the study. However, because this study was conducted in a single hospital, there were no significant differences in treatment methods. In the case of vitrectomy or evisceration, the initial treatment was intravitreal antibiotics injection, and if the condition worsened despite treatment, the next step was vitrectomy or evisceration, depending on the ocular condition. Despite these limitations, this study’s findings are useful as there has been no report that compared and analyzed the characteristics of and risk factors associated with different types of endophthalmitis over a long study period using a relatively large sample size.

## Conclusion

We compared six types of endophthalmitis based on etiology. Age, sex, initial symptoms, the interval between the primary causable event and diagnosis of endophthalmitis, initial VA, final VA, treatment method, and culture results showed statistically significant differences among the groups. Advanced age and initial VA were identified as the most related risk factors for poor visual prognosis. Therefore, early diagnosis and treatment of endophthalmitis are important, especially in older patients.
